# Evaluating treatment effectiveness: Complementing RCTs with real-world data

**DOI:** 10.1093/ajhp/zxag035

**Published:** 2026-02-09

**Authors:** Christina G Rivera, Essy Mozaffari, Stephanie H Read, Andre C Kalil

**Affiliations:** Department of Pharmacy, Mayo Clinic, Rochester, MN, USA; Medical Affairs, Gilead Sciences, Foster City, CA, USA; Evidence and Access, Certara, London, UK; Division of Infectious Diseases, Department of Internal Medicine, University of Nebraska Medical Center, Omaha, NE, USA

**Keywords:** comparative effectiveness research, feasibility, fitness-for-purpose, real-world data

## Abstract

**Purpose:**

While randomized controlled trials remain the gold standard for assessing treatment efficacy, studies using real-world data (RWD) offer valuable insights into treatment effectiveness across broader, more diverse patient populations. This commentary explores the importance of using fit-for-purpose data and emphasizes the need for rigorous evaluation of RWD quality to support valid and actionable evidence generation.

**Summary:**

The utility of RWD-based studies depends heavily on the fitness-for-purpose of the data source, which requires careful assessment of 5 key quality dimensions: relevance, extensiveness, timeliness, coherence, and reliability. Practical examples from coronavirus disease 2019 (COVID-19) comparative effectiveness research are used to illustrate each data quality domain.

**Conclusions:**

As the need for RWD increases, especially for post–COVID-19 pandemic decision-making, ensuring high data quality and appropriate study design is critical. Proper evaluation of RWD sources enhances the credibility of findings and supports their use in meta-analyses, clinical guidelines, and healthcare policy.

Key Points

- Ensuring real-world data (RWD) are fit-for-purpose requires assessing 5 key quality dimensions: relevance, extensiveness, timeliness, coherence, and reliability.
- Evaluating the fitness-for-purpose of data involves confirming the RWD source captures accurate, complete, and relevant information on exposures, outcomes, covariates, and the study population to support valid comparative effectiveness research.
- RWD-based studies are increasingly essential for evaluating COVID-19 treatment effectiveness, especially as large-scale randomized controlled trials become less feasible in the endemic phase.

The evaluation of treatment efficacy and effectiveness requires a robust interpretation of the totality of evidence from diverse study designs, including randomized controlled trials (RCTs), meta-analyses and observational studies (eg, cohort studies, case-control studies) utilizing real-world data (RWD). RCTs are considered the gold standard for establishing treatment efficacy, yet they may face feasibility and generalizability constraints.^1^ Meta-analyses aggregate findings from multiple studies, offering thorough insights from RCTs and/or studies using RWD (hereafter, RWD-based studies) that employ different methodologies. While meta-analyses can provide high-level evidence summaries, the robustness of their conclusions depends on the quality and homogeneity of the underlying data.^2^

RCTs primarily assess treatment efficacy (ie, whether a treatment works), whereas RWD-based studies can provide insights into comparative effectiveness, evaluating how well treatments perform in real-world settings relative to available therapeutic options. They can provide complementary insights into treatment effectiveness across diverse patient populations, delineating treatment effects in routine clinical care and offering evidence on long-term outcomes in large populations, including patient subgroups often excluded from or poorly represented in RCTs.^3,4^

Inherent but well-documented limitations of RWD-based studies, including susceptibility to bias and confounding (summarized in Table 1),^5^ call for highly principled approaches to their implementation. The first step in conducting an RWD-based study is to articulate a concise, unambiguous research question that is clinically or scientifically meaningful and clearly specifies the population, intervention, comparison, and outcome of interest.^6^ Second, RWD are collected outside the controlled environment of clinical trials, originate from diverse sources, and often lack standardized data collection protocols, making them more susceptible to missing, inaccurate, or inconsistent data. Therefore, data quality assessments are essential to ensure the use of fit-for-purpose data to address the research question. Third, given the limitations of these data, appropriate and rigorous methodological strategies should be prespecified and used to mitigate bias, handle missing data, and evaluate the robustness of the findings.^3,5,7^ Without principled study design and careful analysis, RWD can reveal associations but cannot contribute credible evidence towards establishing causality. The evolution of coronavirus disease 2019 (COVID-19) towards endemicity underscores the need for evolving and contemporary evidence paradigms with the increasing reliance on RWD to support meta-analyses and inform clinical guidelines, healthcare providers, and formulary decision-makers.^8,9^ While several seminal RCTs informed early therapeutic strategies,^10–13^ further large-scale RCTs in the postpandemic phase are improbable, shifting the reliance to RWD-based studies to establish treatment effectiveness during the endemic phase and among key subgroups, including the immunocompromised.^14^ Important evidence regarding the effectiveness of severe acute respiratory syndrome coronavirus 2 (SARS-CoV-2) therapeutics has come from electronic healthcare records (EHRs), claims databases, and disease registries.^15–21^

**Table 1.** Summary of Sources of Bias and Confounding in Comparative Effectiveness Research

Category	Definition	Subtypes description	Subtype definition
**Selection bias**	Study population differs systemically from the target population	Ascertainment bias	The method of identifying or recruiting the study population makes some members of the target population less likely to be included than others
		Attrition bias	Systematic differences between treatment groups caused by differential loss to follow-up, where participants who leave the study differ from those who remain
		Survivorship bias	Study population excludes people who did not survive long enough to receive or be observed on treatment, leading to overestimates of treatment effect
**Information bias**	Distortion in measure of association caused by a lack of accurate measurements of key study variables	Misclassification bias	Treatments or outcomes are incorrectly classified, resulting in biased estimates of the association between treatment and outcome
		Detection bias	Outcomes are more likely to be identified or reported in one treatment group than in the comparison group
**Confounding**	Distortion of the estimated effect of a treatment on an outcome caused by a third variable associated with both the treatment and the outcome	Confounding by indication	The reason for receiving a specific treatment is itself a true cause of the outcome, leading to a distorted observed association
		Residual confounding	Confounding that remains after attempts to adjust for measured confounders due to incomplete control or imperfect measurement
**Time-related bias**	Systematic errors that arise when the timing of treatment initiation, follow-up, or outcome measurement is not handled appropriately in a study	Immortal time	A period during which the outcome cannot occur is incorrectly attributed to the treatment group, biasing the effect estimate
		Time-lag bias	Treatment groups begin therapy at different stages of disease, making comparisons of outcomes inaccurate

Given the aforementioned limitations of RWD-based studies, enhanced literacy in interpreting RWD-based research is required to distinguish credible findings from unreliable and potentially misleading results. A key element of study appraisal is understanding the data quality and fitness-for-purpose of the chosen RWD source for use in estimating treatment effectiveness. According to the European Medicines Agency (EMA) Data Quality Framework, the 5 main determinants of data quality are data relevance, extensiveness, timeliness, coherence, and reliability.^22^ To support the evaluation and interpretation of RWD-based studies, this commentary describes the 5 key determinants of data source quality and fitness-for-purpose with examples taken from COVID-19 comparative effectiveness research.

## Data relevance

Data relevance refers to whether the data source captures the necessary elements to address the research question. Specifically, the data source should contain data that allows the accurate identification of exposures (eg, SARS-CoV-2 treatments), relevant outcomes, key covariates, and study population to enable the generation of robust evidence. The following subsections describe the importance of the RWD source capturing these key study elements and the potential implications of failing to do so.

### Availability of exposure data

The availability of precise and consistent identification of exposures, specifically administered treatments, is fundamental to robust comparative effectiveness research.^23^ This includes distinguishing between specific therapeutic agents and combinations of treatments, as well as accurately recording the administration date and dosage. Such granularity is essential to avoid misclassification bias, which can arise if treatments are incorrectly categorized or inaccurately recorded.^23,24^ The RWD source should provide precise information relating to the timing of treatment administration to enable the temporal relationship between the exposure and endpoint to be established, thereby supporting causal inference. Additionally, precise temporal data facilitate the analysis of time-sensitive effects, such as whether early or delayed treatment administration influences outcomes differently. This is particularly important for conditions such as COVID-19, where the timing of interventions relative to disease progression can significantly impact their effectiveness.

### Availability of outcome data

A robust comparative effectiveness study must focus on outcomes that are clinically relevant, patient centered, and reliably recorded in the data source. Hospitalization, intensive care unit admissions, supplemental oxygenation requirement (eg, mechanical ventilation), and mortality are some of the key outcomes for evaluating treatment effectiveness in the context of COVID-19.^25^ Ideally, the definition of the outcome should be explicitly described in published studies to enable the assessment of the likelihood of misclassification bias and the objective interpretation of the reliability of the findings.^23^ Optimally, validated outcome definitions should be employed to minimize the risk of misclassification. In assessing comparative effectiveness studies, attention should be paid to nuances in the capture of these outcomes. For example, many administrative databases may not capture mortality events occurring following discharge or outside of a healthcare setting, therefore necessitating the use of in-hospital mortality as a proxy or linkage to alternative, complementary RWD sources. Similarly, intensive care unit admissions can also be impacted by hospital-specific protocols and may evolve over time, as was the case in the COVID-19 pandemic during surges.^26^

### Availability of covariate data

The capture of covariates such as patient demographics, comorbidities, and clinical severity is an essential component of RWD-based studies since these covariates can be used to balance patient characteristics across comparator groups to reduce the substantial risk of selection bias and confounding in RWD comparative effectiveness studies, an approach that is analogous to randomization in RCTs.^5^ In the absence of random allocation of treatments in real-world studies that utilize routinely collected data, comparison groups are likely to differ in key characteristics since patients are administered treatments by their healthcare providers primarily based on characteristics such as disease severity, risk of progression, age, and vulnerability. This can lead to confounding by indication and necessitates the application of additional analytic steps.^24^ Confounding by indication is a form of bias that complicates the interpretation of treatment effects and obscures the true relationship between the treatment and the outcome of interest, potentially leading to inaccurate conclusions.^5^ It occurs when patients receive a treatment based on their underlying risk of the outcome, rather than randomly, so that the factors influencing treatment choice are also related to the outcome. For instance, in comparing the effectiveness of corticosteroids and antivirals in reducing the risk of mortality among hospitalized patients with COVID-19, confounding by indication must be addressed since corticosteroids are recommended for critically ill patients experiencing severe inflammatory dysregulation.^27^ These patients, by virtue of their clinical presentation, typically have a more severe form of the disease and a worse prognosis than patients receiving antivirals, who are often at earlier stages of disease progression.^27,28^ As a result, differences in mortality may reflect baseline severity rather than treatment effectiveness.

When patient characteristics differ between treatment groups, statistical methods like propensity score matching and inverse probability of treatment weighting (IPTW) can reduce confounding by utilizing observed covariate data to balance groups.^29–31^ Propensity scores reflect the probability of being administered the treatment of interest, conditional on measured baseline covariates, and can be used to match patients or create weighted samples that balance covariates between the two or more groups.^31^ IPTW retains the full sample by weighting all individuals based on their propensity scores, while propensity score matching reduces the sample size by discarding unmatched patients.^30^ Nonetheless, it is important to note that the aforementioned methods can adjust for observed covariates only and that alternative methods such as instrumental variable analysis are required to account for confounding by unmeasured confounders.^7^

For comparative effectiveness studies of SARS-CoV-2 therapeutics, no single data source is likely to capture all covariates that may confound the relationship between treatments and outcomes. The most appropriate source is therefore the one that best satisfies the minimum requirements for capturing critical covariate information, with any constraints clearly documented as limitations. This includes ensuring adequate capture of variables such as baseline oxygen needs, age, comorbidities, clinical status, and concurrent medications in COVID-19 studies.^27,28,32,33^ Where such covariates are not directly available, the availability of appropriate proxies should be explored.

### Availability of data to define the study population

To avoid selection biases, the data source should hold data for individuals from the target population. For example, in comparative effectiveness studies of SARS-CoV-2 treatments administered in hospital settings, the source should allow for the accurate identification of patients hospitalized *for* COVID-19 rather than *with* COVID-19. This may be achieved in some sources through the inclusion of patients with a primary International Classification of Diseases, 10th Revision, Clinical Modification (ICD-10-CM) diagnosis code of COVID-19 rather than a secondary diagnosis or an indicator that the condition was “present on admission.” However, differences in diagnosis coding practices, as applicable to the data source being used, should also be considered. Depending on the data source, a primary diagnosis in an inpatient setting describes the underlying diagnosis that was the most serious and/or resource intensive,^34^ while a principal diagnosis describes the condition primarily responsible for the admission of the patient, which is established after diagnostic evaluation.^35^ Including patients who were hospitalized *with* COVID-19 in comparative effectiveness studies may introduce a selection bias to the findings since these patients will be managed differently from those patients hospitalized *for* COVID-19 and may also have particularly heterogeneous prognoses. The study population should therefore be well described to allow for the assessment of the risk of such selection biases. Selecting a data source that accurately represents the target population is crucial for ensuring that study findings are generalizable and have strong external validity, ensuring findings are applicable beyond the specific study population.

### Data extensiveness

Data extensiveness relates to the coverage and completeness of an RWD source.^22^ In appraising comparative effectiveness studies, careful consideration should be given to whether the source provides data for a patient population that are representative of the target population.^5^ As an example, single-center databases capture data for a specific group of patients, determined by the hospital’s location, resources allocation, local treatment protocols, and population case mix. Accordingly, these patients are likely to have different characteristics, treatments, and outcomes than patients from other hospitals. Findings from single-center studies are therefore less generalizable than studies utilizing RWD covering multiple hospitals.

Given that comparative effectiveness studies of SARS-CoV-2 therapeutics typically assess increasingly rare outcomes, such as hospitalizations and deaths, fit-for-purpose RWD sources need to capture data for thousands of eligible patients to enable the detection of clinically meaningful differences in the rates of relevant outcomes across treatment groups.^18,19,36^ For this reason, single-center studies that aim to examine treatment effectiveness may have limited population representativeness or statistical power to generate meaningful or robust evidence. In the absence of data for many thousands of patients, RWD-based studies should justify their choice of sample size through the detailed description and provision of a power estimation for detecting a predefined treatment effect size.^37^

### Data timeliness

Data timeliness refers to the availability of the data at the relevant time for evidence generation and the ability to access and perform analyses within an acceptable timeframe.^22^ This dimension of data quality was particularly pertinent during the early phase of the pandemic, when there was a need for rapid evidence generation during a period characterized by rapid evolution in treatment protocols and knowledge of the disease that necessitated expedited access to contemporaneous data. Although the urgency for evidence generation has changed in the current endemic phase with the availability of vaccines and widespread immunity, it remains essential for future COVID-19 research to employ RWD that supports the production of timely and accurate evidence reflecting present-day treatment effectiveness amid the emergence of evolving severe acute respiratory syndrome coronavirus variants. For instance, when evaluating the effectiveness of a treatment of interest in comparison to the standard of care, it is imperative that the standard of care treatments captured by the RWD source represent current clinical practices to ensure the findings remain relevant to contemporary medical contexts.

### Data coherence and data reliability

Data coherence and data reliability are further key dimensions of data source quality. Data coherence relates to consistency in the way different parts of an overall dataset are represented (eg, using standardized coding systems or standardized formatting).^22^ For example, data coherence issues may arise when the administration of supplemental oxygenation is not uniformly captured across hospitals contributing to claims databases, with some hospitals capturing the use of supplemental oxygenation on an itemized basis and others including it within room charges. Inconsistencies can create problems for the analysis by introducing misclassification biases. The EMA defines data reliability as the closeness of the data in the dataset relative to the observed reality.^22^ For example, some COVID-19 studies that used ICD-10 diagnosis codes to identify cases may have been influenced by variability in hospitals’ adoption of the specific COVID-19 diagnosis code, particularly during the early period following its introduction. Consequently, case identification may have lacked reliability; however, available evidence suggests that the extent of this issue was likely limited.^38^ Both dimensions are critical in all RWD-based studies to enable the generation of unbiased and unconfounded estimates.^22^ A complete description of all methods used should be published to ensure findings are reproducible, and any concerns regarding the consistency or accuracy of data recording should be clearly acknowledged in the study’s limitations. Additionally, potential biases that could arise due to the underlying funding sources for a study should be carefully considered, and validation of the findings through independent verification or replication of the study could help safeguard appropriate interpretation of the findings.

## Conclusion

Ensuring that RWD-based studies produce reliable and actionable evidence requires careful assessment of the fitness-for-purpose of the data and critical appraisal of the methods and the findings. Evidence generated from RWD-based studies should be carefully evaluated before being included in meta-analyses or being used to inform clinical guidelines, inform clinical or hospital protocols, and support healthcare provider decision making. As the COVID-19 endemic phase continues, RWD-based studies will remain essential for evaluating treatment effectiveness, particularly when large-scale RCTs are not feasible.

## Data Availability

No new data were generated or analyzed in support of this article.

## Additional information

The authors provided the following information regarding their contributions to this work: All authors contributed substantially to the manuscript and agreed to the final submitted version.
